# Awareness and knowledge of Human Papillomavirus (HPV) infection among high-risk men of Hispanic origin attending a Sexually Transmitted Infection (STI) clinic

**DOI:** 10.1186/1471-2334-12-346

**Published:** 2012-12-12

**Authors:** Vivian Colón-López, Ana P Ortiz, Lizbeth M Del Toro-Mejías, Hermes García, Michael C Clatts, Joel Palefsky

**Affiliations:** 1 Department of Health Services Administration, Graduate School of Public Health, University of Puerto Rico, PO BOX 365067, San Juan, 00936-5067, Puerto Rico; 2 Cancer Control and Population Sciences Program, Puerto Rico Comprehensive Cancer Center, University of Puerto Rico, 89 De Diego Ave. Suite 105m, San Juan, 00927-6346, Puerto Rico; 3 Department of Biostatistics and Epidemiology, Graduate School of Public Health, University of Puerto Rico, PO BOX 365067, San Juan, 00936-5067, Puerto Rico; 4 Latin American Center for Sexually Transmitted Diseases, Puerto Rico Department of Health, San Juan, 00927, Puerto Rico; 5 Department of Medicine, UCSF, San Francisco, CA, 94143-0126, San Francisco

**Keywords:** HPV, HPV knowledge, Men, Puerto Rico

## Abstract

**Background:**

Genital Human papilloma virus (HPV) is one of the most commonly diagnosed Sexually Transmitted Infection (STIs) in men and women. Knowledge about HPV infection among men is limited. This study aims to determine correlates of adequate knowledge of HPV infection among men who attend an STI clinic in Puerto Rico.

**Methods:**

A cross-sectional study of 206 men was conducted at an STI clinic in San Juan, PR. Adequate knowledge was defined as a score of at least 70% of correct responses among those men who reported having ever heard of HPV. Variables that achieved statistical significance in the bivariate analysis (p<0.05) were included in the multivariate logistic regression model.

**Results:**

Although 52.5% of men reported having heard of HPV infection before the survey, only 29.3% of this sub-group had an adequate knowledge of HPV. Most men did not know that HPV is a risk factor for anal (38.7%), penile (50.0%) and oral (72.6%) cancer. Factors associated with adequate knowledge of HPV in age-adjusted models were being men who have sex with men (MSM) (OR=2.6;95%CI=1.1-6.1), self-report of genital warts (OR=3.2;95%CI=1.3-7.9) and herpes (OR=7.4;95% CI=2.2-25.1). MSM was marginally associated with adequate knowledge (OR=2.3;95% CI=0.9-5.9) and self-report of herpes remained significantly associated (OR=5.0;95%CI=1.3-18.4) in multivariate logistic regression analysis.

**Conclusions:**

Awareness and knowledge of HPV was very low in this group of men. Interventions to increase knowledge and awareness in this group are necessary to promote preventive practices for HPV-related cancers in high-risk groups.

## Background

Human papilloma virus (HPV) is one of the most commonly diagnosed STIs, with over 630 million HPV infections worldwide and an overall prevalence between 9-13% [1]. A systematic review documented that the prevalence of HPV infection varies among men [2]. However, these wide estimates have been attributed to different sampling methods [2,3], as well as to differences in the populations studied [3]. Regarding HPV-related cancers, worldwide studies have reported presence of HPV infection for oropharyngeal [4], anal [5] and penile cancers [6]. Despite the reported global burden of HPV infection and HPV-related cancers in men [7], few studies have evaluated HPV and HPV-related cancer knowledge [8,9] particularly among high-risk men.

Although knowledge is not a direct predictor of health behavior [10], it is mediated by attitudes, risk perceptions, social influence and self-efficacy [11]. Thus, acquiring knowledge is a key first step to the success of any health education intervention. Although there has been increased attention to HPV awareness and knowledge in the literature [12,13], most of the available research has focused on women [11,14,15]. Studies in men, particularly among those of Hispanic origin, are scarce. Among the few studies that have evaluated gender differences, men have lower knowledge about HPV compared to women [16,17].

In Puerto Rico (PR), despite the HIV epidemic and documented high-risk sexual practices in population-based studies [18,19], there is limited epidemiological and behavioral research on knowledge of HPV infection, particularly among high-risk groups; this may delay the development of effective interventions to reduce the burden of HPV infection. This kind of research is necessary, given that epidemiological evidence indicates that men in PR, particularly HIV+ men, have higher rates of HPV-related cancers compared to the general population of PR [20]. Therefore, in order to understand the determinants of knowledge of HPV infection, it is necessary to evaluate high-risk groups, particularly individuals whose average rate of acquisition of new sexual partners is sufficiently high to maintain increasing numbers of secondary infections. STI clinics can be an ideal setting for reaching these high-risk groups [21]. Studies in STI clinics have reported high prevalence estimates of HPV infection; but knowledge in patients from these settings is limited [22-24]. This study aims to describe the proportion of men that attend an STI clinic in PR who are aware of HPV, determine their level of knowledge about HPV infection, and identify correlates of HPV knowledge within this group.

## Methods

This study was part of an ongoing epidemiological core study among men and women attending the Latin American Center for Sexually Transmitted Diseases (CLETS, by its initials in Spanish), located in San Juan, Puerto Rico A full description of the methodology used in the core study has been described elsewhere [25]. Briefly, it involved recruitment of men and women from the clinic waiting, coupled with subsequent screening to confirm eligibility (at least 16 years of age) and capacity for informed consent. Study participation was voluntary and written informed consent was obtained. Study subjects participated in a structured behavioral interview that was administered by the research staff and data were recorded utilizing QDS software [26]. Domains in the survey included demographic characteristics (age, education, income, employment status, and marital status); number of female sexual partners (last 90 days), sexual identity (Men who have Sex with Men (MSM), non-MSM), consumption of alcohol, tobacco or illegal substances (last 90 days); sexual history and self-reported history of STIs; and current sexual risk profile (last 90 days).

Following completion of the core survey interview, all male participants were informed of the opportunity to participate in an additional interview as part of an HPV study. Men expressing interest in the HPV study were required to provide additional written informed consent for a second interview, which included questions regarding HPV awareness, HPV knowledge, perceived susceptibility to HPV-related cancers, HPV vaccine awareness, and willingness to be vaccinated against HPV.

Data collection procedures were approved by the University of Puerto Rico, Medical Sciences Campus Institutional Review Board (UPR-IRB).

### HPV knowledge

Based on both existing measures [27] and additional input from the study team, a 21-item scale measure was used to address HPV knowledge. Response options included true, false and don’t know. An incorrect or *‘don’t know’* answer was given a score of 0. An example of items included in this scale was ‘*only women can get infected with HPV*’. Latter sets of questions were assessed only among those who reported having heard of HPV infection before the survey (n=106), or 52.5% of the total sample.

### Statistical analysis

Exploratory factor analysis of the 21 item scale measuring HPV knowledge identified 4 subscales, in which one factor (15 item scale) was related to HPV knowledge. Chrombach alpha for the 15 item scale was 0.75.

A mean knowledge score was computed by summing correct responses of the 15 item scale, with higher scores meaning a higher knowledge. Adequate knowledge was defined as a minimum score of 70% [28]. Chi-square tests were used to identify relationships between demographic and behavioral characteristics, and self-reported STIs with adequate HPV knowledge. Variables that achieved statistical significance (p < 0.05) were assessed in the logistic regression models. Due to the reported association between age and HPV knowledge in the literature [29], Model 1 was adjusted for age. Then, a multivariate logistic regression model was performed (Model 2) to determine predictors of adequate HPV knowledge after controlling by all the independent variables in the model. All statistical analyses were performed using the statistical package SAS (Version 9.1,Cary,NC).

## Results

### Participants’ characteristics

A total of 206 men participated in the study, of which 202 (98.1%) completed the full assessment (behavioral interview and HPV supplement). Demographic and individual HPV risk-related characteristics are shown in Table 1. The mean age of the study participants was 37.8 years (SD=13.12). Over half (54.4%) had less than high school education; 61.9% were employed at the time of the interview. Nearly 20% were without medical insurance (neither private nor government-sponsored plan).

**Table 1:** Factors associated with adequate HPV knowledge among men aware of HPV, attending an STI clinic in Puerto Rico

**Variables**		Adequate HPV Knowledge^*^(n=106)		
	**Total (%) n=202**	**No (%)**	**Yes (%)**	p-value^**^
**Sociodemographic characteristics**				
**Age (years)**				0.969
Mean ± SD	37.8 ± 13.1	36.6 ± 13.2	41.3 ± 17.4	
**Educational Level**				0.607
< High School	36 (17.8)	9 (12.0)	3 (9.7)	
High School	74 (36.6)	28 (37.3)	9 (29.0)	
> High School	92 (45.5)	38 (50.7)	19 (61.3)	
**Employment**				0.915
No	77 (38.1)	21 (28.0)	9 (29.0)	
Yes	125 (61.9)	54 (72.0)	22 (71.0)	
**Individual Annual Income**				0.572
None	57 (28.2)	16 (21.3)	5 (16.1)	
< $15,000	104 (51.5)	40 (53.3)	20 (64.5)	
≥ $15,000	41 (20.3)	19 (25.3)	6 (19.4)	
**Health Insurance**				0.788
None	37 (18.3)	13 (18.1)	4 (13.8)	
Public	119 (58.9)	16 (22.2)	8 (27.6)	
Private	39 (19.3)	43 (59.7)	17 (58.6)	
**Behavioral Factors**				
**Tobacco Use (last 90 days)**				0.253
No	96 (47.5)	32 (42.7)	17 (54.8)	
Yes	106 (52.5)	43 (57.3)	14 (45.2)	
**Alcohol Use (last 90 days)**				0.812
No	82 (40.6)	26 (34.7)	10 (32.3)	
Yes	120 (59.4)	49 (65.3)	21 (67.7)	
**Drug Use (last 90 days)**				0.132
No	128(63.4)	44 (58.7)	23 (74.2)	
Yes	74(36.6)	31 (41.3)	8 (25.8)	
**Female Sexual Partners (last 90 days)**				0.589
None	100 (49.5)	37 (49.3)	17 (54.8)	
1 partner	77 (38.1)	28 (37.3)	12 (38.7)	
> 1 partner	25 (12.4)	10 (13.3)	2 (6.5)	
**Sexual Identity**	0.028			
Non-MSM	142 (70.3)	51 (68.0)	14 (45.2)	
MSM	59 (29.2)	24 (32.0)	17 (54.8)	
**Lifetime history of STIs (self-reported)**				
**Herpes**				0.001^**^
No	171 (84.7)	70 (93.3)	21 (67.7)	
Yes	30 (14.9)	5 (6.7)	10 (32.3)	
**Gonorrhea**				0.120
No	170 (84.2)	65 (86.7)	23 (74.2)	
Yes	31 (15.3)	10 (13.3)	8 (25.8)	
**HIV**				0.913
No	118 (58.4)	42 (56.0)	17 (54.8)	
Yes	84 (41.6)	33 (44.0)	14 (45.2)	
**Genital Warts**				0.009
No	156 (77.3)	58 (77.3)	16 (51.6)	
Yes	45 (22.3)	17 (22.7)	15 (48.4)	
**Chlamydia**				1.000^**^
No	179 (88.6)	68 (90.7)	28 (90.3)	
Yes	21 (10.4)	7 (9.3)	3 (9.7)	
**Syphilis**				0.530
No	168 (83.2)	62 (82.7)	24 (77.4)	
Yes	33 (16.3)	13 (17.3)	7 (22.6)	

^*^Only those that reported having heard about HPV were included in this analysis. Adequate knowledge was defined as a score of at least 70% of the correct responses.

^**^Fisher’s exact tests were performed.

### Recent tobacco, alcohol and drug use

More than half reported recent (last 90 days) use of tobacco (52.5%) and alcohol (59.4%). More than a third (36.6%) reported recent illicit drug use.

### Sexual behaviors and practices

12.4% of the men interviewed have had more than one sexual partner in the last 3 months. A significant proportion of study participants identified as being men who have sex with men (MSM) (29.2%). Among those reporting vaginal sex, more than a half (56.9%) reported condom use in their last sexual intercourse, and 33.7% of the participants were circumcised (*data not shown*).

### HPV knowledge

More than half (52.5%; n=106) of the sample had heard about HPV infection before the survey. Primary sources of information included television (24%), print media (e.g., magazines, newspapers, etc.) (13%), and health care professionals (10.5%) (*data not shown*). However, only 29.3% of these men demonstrated an adequate level of knowledge of HPV. Table 2 shows the absolute and relative frequencies of correct answers for each item scored regarding HPV knowledge. The proportion of the correct responses ranged from 16.0% to 90.6%. Most knew that HPV is a sexually transmitted infection (85.9%). However, more than half 58.5% were unaware that genital HPV is transmitted through skin-to-skin contact. With respect to knowledge of the long-term consequences of HPV infection, half (50.0%) were aware that HPV can cause penile cancer and just 27.4% knew that HPV infection has a role in oral cancer. Conversely, 38.7% of men were unaware of the link between HPV and anal cancer.

**Table 2:** Absolute and relative frequencies of correct answers for each item scored regarding HPV knowledge (n=106)

**Item**	**Frequency**	**% Correct**
1. Genital warts are caused by HPV	70	66.04
2. HPV is a virus that can cause cervical cancer	82	77.36
3. I can infect my sexual partner with HPV, even though I don’t present the symptoms	86	81.13
4. HPV can cause genital warts in the penis	60	56.60
5. HPV can cause genital warts in the anus	55	51.89
6. HPV can cause penile cancer	53	50.00
7. HPV is sexually transmitted	91	85.85
8. As more sexual partners I get, more the risk of getting infected with HPV	96	90.57
9. There are many HPV types	63	59.43
10. HPV is associated with oral cancer	29	27.36
11. Some HPV types can disappear without treatment	32	30.19
12. Genital HPV is transmitted through skin by skin contact	44	41.51
13. Condom use prevents HPV infection^¥^	63	59.43
14. All HPV types cause cancer^¥^	47	44.34
15. HPV can cause anal cancer	65	61.32

Mean Score ± SD: 8.8 ± 3.5 points.

^¥^Items 13 and 14 were false.

Table 1 shows the association between demographic, behavioral and clinical factors associated with adequate knowledge of HPV. Factors significantly associated with adequate HPV knowledge in bivariate analyses were being MSM (p < 0.028), having a self-reported history of herpes infection (p < 0.001), and a self-reported history of genital warts (p < 0.009).

Table 3 shows the results of the age-adjusted and multivariate logistic regression models evaluating factors associated with adequate HPV knowledge among men.

**Table 3:** Logistic regression models of factors associated to adequate HPV knowledge

	**Model 1**^**†**^		**Model 2**^**‡**^	
**Variables**	**OR (95% CI)**	**P-value**	**OR (95% CI)**	**P-value**
**Sexual Identity**		0.030		0.070
Non-MSM	1.00		1.00	
MSM	2.59 (1.10-6.11)		2.34 (0.93-5.88)	
**Herpes**		0.001		0.016
No	1.00		1.00	
Yes	7.38 (2.17-25.06)		4.97 (1.34-18.41)	
**Genital warts**		0.010		0.121
No	1.00		1.00	
Yes	3.22 (1.32-7.85)		2.19 (0.81-5.91)	

^**†**^**Model 1:** Adjusted by age.

^**‡**^**Model 2:** Adjusted by age, sexual identity, self-reported history of herpes and genital warts.

In the age-adjusted model, men who have identified as MSM were almost three times more likely to have an adequate knowledge of HPV compared to Non-MSM (age-adjusted OR=2.59; 95% CI=1.10-6.11). Men who reported a history of herpes (age-adjusted OR=7.38; 95% CI=2.17-25.06) and genital warts (age-adjusted OR=3.22; 95% CI=1.32-7.85) also were more likely to have adequate knowledge of HPV. In multivariate models (Table 3), only having a history of herpes infection remained positively associated with adequate HPV knowledge. MSM were up to two times more likely to have an adequate knowledge of HPV compared to non-MSM. However, this association achieved only marginal significance (p < 0.07).

## Discussion

To our knowledge, this is the first study to attempt to examine awareness of HPV infection among men in PR. In a sample in which none of the study participants has being vaccinated and the awareness of the vaccine is low [30], results show that despite acknowledgement by more than half of the sample that they had heard about HPV infection, a low proportion of study participants had adequate knowledge of HPV. It should be pointed out that among those men that were aware of HPV infection, high levels of knowledge regarding the association of HPV infection with cervical cancer and some risk factors (such as number of sexual partners) was reported. It is likely that the higher levels of adequate HPV knowledge among men with a self-reported history of STIs stems from education they received during the course of STI treatment. This suggests that STI service providers are a good source of education and also that HPV knowledge may be retained in this high-risk population, although further research in this area is warranted. Our results indicate that greater attention is needed to educate men about the potential long-term consequences of HPV infection, including increased risk for oral, penile, and anal cancers [31].

Common sources of HPV information that were identified in this study were television, magazine and newspaper, followed by health care professionals and physicians. These sources of information are inconsistent with results from the population-based 2009 HINTS survey in PR, which showed that the Internet is the primary source for cancer information used by the general population of PR [32]. A possible explanation for this discrepancy is that our study sample was recruited in an STI clinic and primarily comes from a low socioeconomic background. Thus, sources of information and health literacy in this group might differ to those from the general population. This needs to be further investigated in order to identify a mechanism for dissemination which will help to increase knowledge about HPV in this high-risk population.

After age-adjusted logistic regression analysis, a marginal association between MSM and adequate knowledge of HPV was observed. Findings from an online survey in the US show similar results [31]. In this study, 22% of MSM reported genital warts; therefore, knowledge of HPV infection might have been the result of their previous infection. Additionally, qualitative studies among MSM in Puerto Rico attending STI clinics have shown that knowledge of HPV-related cancers in this group is limited [33]. Since studies have demonstrated a high prevalence of HPV infection in MSM, along with a high rate of HPV-related anal cancer [34], this group constitutes a unique population that will greatly benefit from the development of health interventions to increase knowledge of HPV and of related morbidities.

Although results from this clinic-based study are of great relevance for the development of cancer prevention and control efforts among men in PR, our findings need to be interpreted with caution. Research in an STI setting has limitations. Primary among these is that our findings might not generalize to the general population. Specificity and complexity of some of the items of our HPV knowledge score also needs to be further explored for future efforts. Despite its potential limitations, data collected from a highly selective population, such as a public STI clinic, may also offer an opportunity to reach targeted high-risk groups more efficiently.

## Conclusion

In summary, this study highlights the low prevalence of HPV awareness and of adequate knowledge of HPV infection in a sexually active group of men attending an STI clinic in Puerto Rico. Findings highlight the need to increase HPV awareness, primarily of HPV-related cancers such as oral and anal cancer. STI clinics might serve as venues for cancer prevention and control among high-risk men. In order to effectively moving forward strategies in these settings, further research which will help us understanding levels of health literacy, stratified by groups of interest, needs to be performed.

## Competing interests

The authors declare that they have no competing interests.

## Authors’ contributions

VCL worked on the conceptualization of the study, study design, data analysis and interpretation and manuscript preparation; APO worked on the study design, data interpretation and manuscript preparation; LMDT worked on data analysis, interpretation and manuscript preparation; JP worked on conceptualization of the study, manuscript preparation and interpretation of data; MCC worked on the study design; HG worked on conceptualization of the study, study design and interpretation. All authors read and approved the final manuscript.

## Pre-publication history

The pre-publication history for this paper can be accessed here:

http://www.biomedcentral.com/1471-2334/12/346/prepub

## References

[B1] Pagliusi S Vaccines against Human Papillomavirus World Health Organization http://www.who.int/vaccines/en/hpvrd.shtml (accessed 29 March 2010)

[B2] Dunne EF Nielson CM Stone KM Markowitz LE Giuliano AR Prevalence of HPV infection among men: a systematic review of the literature J Infect Dis 2006 194 1044 1057 10.1086/507432 16991079

[B3] Nielson CM Harris RB Dunne EF Abrahamsen M Papenfuss MR Flores R Markowitz LE Giuliano AR Risk factors for anogenital human papillomavirus infection in men J Infect Dis 2007 196 1137 1145 10.1086/521632 17955431 PMC3877918

[B4] Kreimer AR Clifford GM Boyle P Franceschi S Human papillomavirus types in head and neck squamous cell carcinomas worldwide: a systematic review Cancer Epidemiol Biomarkers Prev 2005 14 467 475 10.1158/1055-9965.EPI-04-0551 15734974

[B5] De Vuyst H Clifford GM Nascimento MC Madeleine MM Franceschi S Prevalence and type distribution of human papillomavirus in carcinoma and intraepithelial neoplasia of the vulva, vagina and anus: a meta-analysis Int J Cancer 2009 124 1626 1636 10.1002/ijc.24116 19115209

[B6] Heideman DA Waterboer T Pawlita M Delis-van Diemen P Nindl I Leijte JA Bonfrer JM Horenblas S Meijer CJ Snijders PJ Human papillomavirus-16 is the predominant type etiologically involved in penile squamous cell carcinoma J Clin Oncol 2007 25 4550 4556 10.1200/JCO.2007.12.3182 17925550

[B7] Giuliano AR Tortolero-Luna G Ferrer E Burchell AN de Sanjose S Kjaer SK Muñoz N Schiffman M Bosch FX Epidemiology of human papillomavirus infection in men, cancers other than cervical and benign conditions Vaccine 2008 26 10 K17 K28 18847554 10.1016/j.vaccine.2008.06.021 PMC4366004

[B8] Reiter PL Brewer NT Smith JS Human papillomavirus knowledge and vaccine acceptability among a national sample of heterosexual men Sex Transm Infect 2010 86 3 241 246 10.1136/sti.2009.039065 19951936 PMC4028225

[B9] Baer H Allen S Braun L Knowledge of human papillomavirus infection among young adult men and women: implications for health education and research J Community Health 2000 25 1 67 78 10.1023/A:1005192902137 10706210

[B10] Viswanath K Breen N Meissner H Moser RP Hesse B Steele WR Rakowski W Cancer knowledge and disparities in the information age J Health Commun 2006 11 1 1 17 16641071 10.1080/10810730600637426

[B11] Tiro JA Meissner HI Kobrin S Chollette What do women in the U.S. know about human papillomavirus and cervical cancer? Cancer Epidemiol Biomarkers Prev 2007 16 2 288 294 10.1158/1055-9965.EPI-06-0756 17267388

[B12] Markham CM Escobar-Chaves SL Addy RC Lewis H Tortolero-Luna G Tortolero SR Knowledge and risk perception regarding HPV among Latino alternative school students in Houston, Texas Tipica 2009 5 1 32 38 21132052 PMC2995251

[B13] Liddon N Hood J Wynn BA Markowitz LE Acceptability of human papillomavirus vaccine for males: a review of the literature J Adolesc Health 2010 46 2 113 123 10.1016/j.jadohealth.2009.11.199 20113917

[B14] Colón-López V Ortiz AP Palefsky J Burden of human papillomavirus infection and related comorbidities in men: implications for research, disease prevention and health promotion among Hispanic men P R Health Sci J 2010 29 3 232 240 20799510 PMC3038604

[B15] Klug SJ Hukelmann M Blettner M Knowledge about infection with human papillomavirus: a systematic review Prev Med 2008 46 2 87 98 10.1016/j.ypmed.2007.09.003 17942147

[B16] Nandwani MC Men's knowledge of the human papillomavirus vaccine Nurse Pract 2010 35 11 32 39 10.1097/01.NPR.0000388900.49604.e1 20975448

[B17] Baer H Allen S Braun L Knowledge of human papillomavirus infection among young adult men and women: Implications for health education and research J Commun Health 2000 25 67 78 10.1023/A:1005192902137 10706210

[B18] Ortiz AP Soto-Salgado M Suárez E del Carmen Santos-Ortiz M Tortolero-Luna G Pérez CM Sexual behaviors among adults in Puerto Rico: a population-based study J Sex Med 2011 8 9 2439 2449 10.1111/j.1743-6109.2011.02329.x 21676177 PMC3474935

[B19] Colón-López V Rodríguez-Díaz CE Ortiz AP Soto-Salgado M Suárez E Pérez CM HIV-related risk behaviors among a sample of men who have sex with men in Puerto Rico: an overview of substance use and sexual practices P R Health Sci J 2011 30 2 65 68 21682148 PMC3119669

[B20] Ortiz AP Pérez J Soto-Salgado M Palefsky J Perez J Colón-López V Burden of Human Papilloma Virus (HPV) related cancers among persons living with AIDS in Puerto Rico 2012 Submitted to MMWR

[B21] Darbes L Crepaz N Lyles C Kennedy G Rutherford G The efficacy of behavioral interventions in reducing HIV risk behaviors and incident sexually transmitted diseases in heterosexual African Americans AIDS 2008 22 10 1177 1194 10.1097/QAD.0b013e3282ff624e 18525264 PMC3749047

[B22] Souza LM Miller WM Nery JA Andrade AF Asensi MD A syphilis co-infection study in human papilloma virus patients attended in the sexually transmitted infection ambulatory clinic, Santa Casa de Misericordia Hospital, Rio de Janeiro, Brazil Braz J Infect Dis 2009 13 3 207 209 10.1590/S1413-86702009000300010 20191198

[B23] Dempsey AF Koutsky LA Golden M Potential impact of human papillomavirus vaccines on public STD clinic workloads and on opportunities to diagnose and treat other sexually transmitted diseases Sex Transm Dis 2007 34 7 503 507 17325621 10.1097/01.olq.0000253337.62932.29

[B24] Svare EI Kjaer SK Worm AM Osterlind A Meijer CJ van den Brule AJ Risk factors for genital HPV DNA in men resemble those found in women: a study of male attendees at a Danish STD clinic Sex Transm Infect 2002 78 3 215 218 10.1136/sti.78.3.215 12238658 PMC1744457

[B25] Clatts MC Rodríguez-Díaz CE García H Vargas-Molina RL Colón-López V Pérez-Rios N Goldsamt L Jovet-Toledo GG Sexually transmitted infections clinics as strategic venues for targeting high-risk populations for HIV research and sexual health interventions P R Health Sci J 2011 30 3 101 108 21932709

[B26] Questionnaire Development System http://www.novaresearch.com/Products/qds/index.cfm.

[B27] Ramirez JE Ramos DM Clayton L Kanowitz S Moscicki AB Genital human papillomavirus infections: knowledge, perception of risk, and actual risk in a nonclinic population of young women J Womens Health 1997 6 113 121 10.1089/jwh.1997.6.113 9065380

[B28] Soto-Salgado M Suárez E Ortiz AP Adrovet S Marrero E Meléndez M Colón HM Albizu C Del C Santos M Torres E Pérez CM Knowledge of viral hepatitis among Puerto Rican adults: implications for prevention J Community Health 2011 36 4 565 573 10.1007/s10900-010-9342-6 21125319 PMC3463938

[B29] Pelullo CP Di Giuseppe G Angelillo IF Human papillomavirus infection: knowledge, attitudes, and behaviors among Lesbian, gay men, and bisexual in Italy PLoS One 2012 7 8 e42856 10.1371/journal.pone.0042856 22905178 PMC3414511

[B30] Colón-López V Del Toro-Mejías L Ortiz AP Tortolero-Luna G Palefsky JM Awareness and willingness of the HPV vaccine among high-risk men attending an STI clinic in Puerto Rico P R Health Sci J 2012 4 227 231 PMC3711188 23844472

[B31] Brewer NT Ng TW McRee AL Reiter PL Men's beliefs about HPV-related disease J Behav Med 2010 33 4 274 281 10.1007/s10865-010-9251-2 20162346 PMC4018629

[B32] Tortolero-Luna G Finney-Rutten LJ Hesse BW Davis T Kornfeld J Sanchez M Moser RP Ortiz AP Serrano-Rodriguez RA Davis K Health and cancer information seeking practices and preferences in Puerto Rico: creating an evidence base for cancer communication efforts J Health Commun 2010 15 3 30 45 10.1080/10810730.2010.522698 21154082

[B33] Colón-López V Ortiz AP Pérez N Pattatucci A Understanding of HPV-related cancers among HIV+ individuals in Puerto Rico. Poster presented at The 27th International Papillomavirus Conference 2011 Berlin, Germany

[B34] Palefsky JM Human papillomavirus-related disease in men: not just a women's issue J Adolesc Health 2010 46 4 S12 S19 10.1016/j.jadohealth.2010.01.010 20307839 PMC2871537

